# Reliability of a Virtual Prosthodontic Project Realized through a 2D and 3D Photographic Acquisition: An Experimental Study on the Accuracy of Different Digital Systems

**DOI:** 10.3390/ijerph16245139

**Published:** 2019-12-16

**Authors:** Luca Lavorgna, Gabriele Cervino, Luca Fiorillo, Giovanni Di Leo, Giuseppe Troiano, Marco Ortensi, Luigi Galantucci, Marco Cicciù

**Affiliations:** 1 Private Practice, Telese Terme 82037, Italy; info@odontosinergy.it (L.L.); giovannidileo@outlook.com (G.D.L.); 2 Department of Biomedical and Dental Sciences, Morphological and Functional Images, University of Messina, 98100 Messina, Italy; gcervino@unime.it; 3 Department of Prosthodontics, University of Foggia, 71100 Foggia, Italy; giuseppe.troiano@unifg.it (G.T.); marco.ortensi90@gmail.com (M.O.); 4 Department of Mechanics and Mathematics Management, University of Bari, 70100 Bari, Italy; luigimaria.galantucci@poliba.it

**Keywords:** dentistry, digital planning, intraoral scanner, digital workflow, prosthodontic, virtual

## Abstract

Aims: The study aims to assess the accuracy of digital planning in dentistry, evaluating the characteristics of different intraoral 3D scanners and comparing it with traditional imaging 2D recording methods. Specifically, using computer aided design (CAD) software and measuring inside CAD software, authors want to verify the reliability of different models obtained with different techniques and machines. Methods: 12 patients that needed aesthetic restorative treatment were enrolled in the study. All the patients underwent recording data of the height and width dental elements 1.1, 1.2, and 1.3 size using different technologies and comparing 2D with 3D methods. A T test was then applied in order to verify whether there was a statistically significant difference between the measurements obtained, comparing the different tools data (Emerald, TRIOS, Photogrammetry and DSS (Digital Smile System)) with the reference values. Results: No significant differences emerged in the measurements made with the different scanners (Trios 3Shape ^®^, Planmeca Emerald ^®^) and photogrammetry. Therefore, what should be underlined regarding the 2D measurements is the speed and simplicity compared to all 3D techniques, so this work can help to better define the field of application and the limits connected to 2D techniques, giving a good window of the technique. Conclusions: The low number of patients is not sufficient to provide statistically significant results, but the digital planning future prospects seem to be promising. This study results highlighted how a photogrammetric scanner for dental arches would only have a much smaller shooting field size and greater accuracy. Despite these considerations, the photogrammetric facial scanner provided excellent results for the measurement of individual teeth, showing a great versatility of use.

## 1. Introduction

The introduction of new restorative materials in dentistry, the current knowledge on the enamel-dentin adhesion method, and the use of the computer as an aid for the aesthetic analysis of the smile are the basis of a change in the dental daily practice. The new clinical approach is not very invasive and is therefore able to replace the “real” patient with a “virtual” one. The goal is to enhance the image of the patient, maintaining the health and respecting aesthetics with a balance between teeth and soft tissues [1,2].

The digital revolution opens the way to the virtual patient representing all the patient’s tissues (bone, teeth, gums, face) in a single 3D model. In this way, it is possible to perform preoperative planning and to evaluate surgical, prosthetic and orthodontic treatments. At the same time, it is possible to physically realize the necessary tools for clinical use in the various branches of dentistry [3].

The analogical workflow in esthetic dental rehabilitation includes various phases: from the impression taken through the use of different plastic materials to the development of the plaster model for the realization of the diagnostic wax-up and for the construction of the mock-up. The patient test and evaluation of the mock-up is fundamental to increase the patient’s understanding of the expected post treatment result. The level of patient satisfaction is related to the consistency of the final product with the mock-up. The accuracy of the mock-up depends in turn on the accuracy of the patient’s stomatognathic apparatus morpho-functional characteristics detection [2,4,5].

A great deal of errors occurs in the different phases of a traditional prosthetic workflow, since this process requires the transfer of two-dimensional and three-dimensional data between different operators. The use of pre-visualization and 3D rendering software is guiding clinicians towards a paradigm shift, respecting the standard of traditional care with a reduction of the operator’s error as a priority [4,5,6].

Commonly, the conventional dental impression registration is a simple procedure. However, it is not always easy for the patients. Several authors documented how the dental impression is recorded as an uncomfortable phase for the patients. It is a high risk that the patient may have a poor inclination to an attitude of compliance towards the dental team. The subjects involved in dental treatment therefore favored comfortable clinical procedures, as well as other factors such as the precision of the procedures, the effectiveness of diagnostic devices, and the clinical experience of the dental team, which were all important elements for the final success [5,6,7,8,9].

Although intra oral scanners IOSs can be considered useful tools for capturing impressions in partially edentulous patients, the scientific literature does not seem to support their use in completely edentulous patients. Hence, there is a need to replace conventional clinical procedures requiring physical contact with the patient with others that reduce the patient’s direct involvement and treatment times—without affecting the precision and aesthetic performance of the final prosthesis. Technological evolution is proceeding fast, and the manufacturing companies release new hardware and software monthly to improve the accuracy of their IOS.

Furthermore, it should be emphasized that there are statistically significant differences in the accuracy of different IOSs, especially in the totally edentulous patients scanning [9,10,11]. By using these tools, a complex of digital data can be obtained and it is possible to have a “virtual patient”. The outcome of the procedure is to replace the real patient in a completely digital workflow aimed at the manufacture of dental prostheses that adapt to the patient’s arches in the most appropriate way. In order to realize this project, the dimensional discrepancies between the real patient and the 2D and 3D virtual project should be checked. A further check regards the comparison between the reliability of the 2D project and that of the 3D project.

The present study therefore aims to verify the reliability of the virtual planning of aesthetic cases, carried out starting from photographic acquisitions of the patient’s face using different technologies able to return on the one hand traditional, two-dimensional images, and from the innovative, three-dimensional representations.

## 2. Materials and Methods

### 2.1. Patient Selection

Twelve patients spontaneously following our observation and requesting a restorative treatment to improve the aesthetic aspect of the dental elements of the II sextant were involved in this study.

All the patients were previously informed of their participation in this study and agreed to sign informed consent. The dental restorative procedures have been performed accordingly to the standards set by the World Medical Association (WMA) with the Helsinki Declaration on Human Experimentation.

The sample of patients included six women and six men between 25 and 35 years. The dental elements involved in the treatment have serious aesthetic defects accordingly to the standard proportion reported in the international literature [5,6,7,8,9,10].

The following inclusion and exclusion criteria have been used during recruiting.

Inclusion criteria:

- Patients requesting restorative treatments.

Exclusion criteria:

- Patients with systemic pathologies;
- Patients with oral pathologies, periodontal or articular disease.

### 2.2. Clinical and Laboratory (CAD) Procedures

A silicone impression of the upper dental arch was detected for each patient. The impression material used was the Express 2 PENTA (3M Espe) polyvinyl siloxane (Pioltello MI, Italy), in the double heavy and light body viscosity, delivered through Pentamix 3 automatic mixer (3M Espe, Pioltello MI, Italy).

From each impression, a physical model was subsequently obtained using Fujirock EP type IV dental stone (GC Europe NV, Tokyo, Japan), mixed under vacuum using a Venturi Tornado effect mixer (Silfradent, Forlì-Cesena, Italy) and inserted into the impression with a specific gypsum vibrator (Renfert, Hilzingen, Germany).

Stone models were scanned inside a 3Shape D1000 laboratory scanner (3shape A/S, Copenhagen, Denmark) obtaining an STL (Standard Triangulation Language) file for each of them. These values are taken as reference parameters for subsequent measurements (sample group).

Furthermore, two optical impressions were acquired for each patient; one by using the Planmeca Emerald ™ intraoral scanner, (Planmeca OY, Helsinki, Finland) and the other with the TRIOS^®^ 3 scanner (3shape A/S, Copenhagen, Denmark). PLY and DCM files were respectively obtained and then converted into STL so that they could be processed and analyzed. These values were collected in the group 1 (Planmeca) and group 2 (3Shape).

At the same time the patients were subjected to digital smile system (DSS) acquisition to the official photographic protocol. The photographic exam was conducted with a Canon 5d mark III full frame reflex equipped with Canon EF 100mm f/2.8L Macro IS USM optics (Canon, Tokyo, Japan) and supported by a tripod (Manfrotto, Vicenza, Italy) placed at a distance of 1.50 m from the face of the patients. The photograph depicts the patient with the aid of labial retractors in order to expose the greatest number of dental elements, which is useful for a correct measurement. The produced files were recorded in a JPEG format. The resulting values were collected in the group 3.

The patients were then subjected to the acquisition of the face by applying the photogrammetric technique. The patients used a target placed on the chest—called a collar—and the calibration glasses of the DSS system. The device used for the photogrammetry of the face is the FaceShape Maxi 6 (Polishape 3D, Bari, Italy), in the Maxi Line version composed of six Canon D2000 reflex cameras equipped with Canon 50 mm f/1.8 STM lenses (Canon, Tokyo, Japan). The file resulting from the photographic processing of the Photoscan Professional Edition software (Agisoft LLC., St. Petersburg, Russia) supplied with the device is in an OBJ format.

### 2.3. Outcome

The various format files were then transferred to the Exocad DentalCAD software Matera (Exocad GmbH, Darmstadt, Germany) and the measurements of the dental elements 1.1, 1.2, and 1.3 were performed. Specifically, two linear distances were measured for each of these dental elements:

- one in the apico-coronal sense, from the most apical point of the gingival parabola, i.e., the gingival zenith up to the incisal edge;
- the other in the mesio-distal direction, at the level of the equator of the dental elements, from the most mesial to the most distal point; i.e., the maximum mesio-distal diameter level.

**Figure A1:**
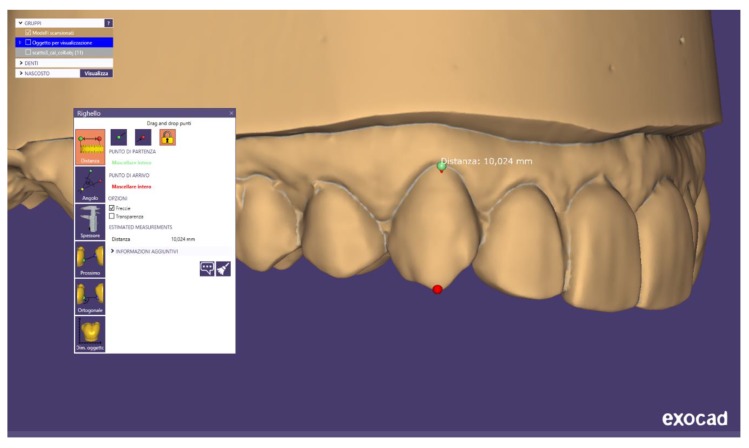
Stone model scan evaluated distance. The sample has been chosen because of its reproducibility and low humidity, and the scan has been used as reference parameters.

**Figure A2:**
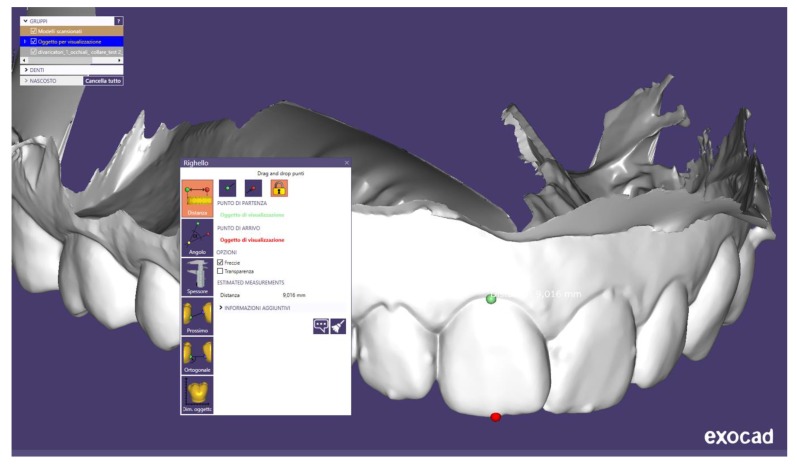
Planmeca Emerald^®^ Scan, distance measure were obtained and compared to stone model.

**Figure A3:**
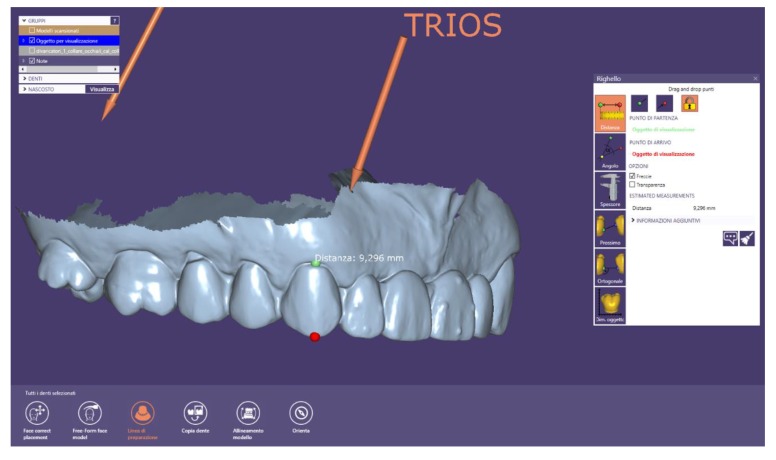
Trios 3Shape scan, measurements of obtained stl file with this scanner were compared to the stone model.

**Figure A4:**
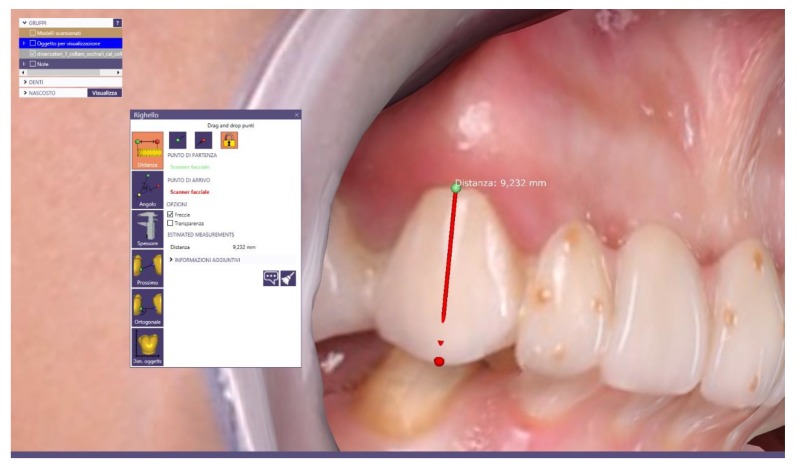
Photogrammetric exam, the device used for the photogrammetry of the face is the Face Shape Maxi 6 (Polishape 3D, Bari, Italy), in the Maxi Line version composed of six Canon D2000 reflex cameras equipped with Canon 50 mm f/1.8 STM lenses.

**Figure A5:**
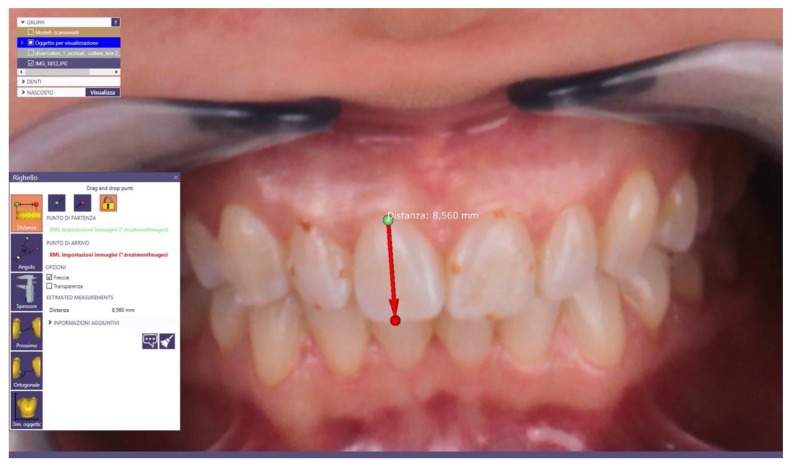
Digital Smile System Exam. Conducted with a Canon 5d mark III full frame reflex equipped with Canon EF 100mm f/2.8 L Macro IS USM optics and supported by a tripod (Manfrotto, Vicenza, Italy) placed at a distance of 1.50 m from the face of the patients.

Precisely, the files imported into the exocad software in order to perform these measurements for each patient are the following:

- 3D model obtained from the scanning in the laboratory of the stone model of the upper arch (figure MODEL IN PLASTER) (Figure A1);
- 3D model of the upper arch created using the Planmeca Emerald intraoral scanner (figure MODEL EMERALD) (Figure A2);
- 3D model of the upper arch acquired with the 3Shape TRIOS intraoral scanner (3shape, Copenhagen, Denmark) (TRIOS MODEL) (Figure A3);
- 3D model of the face detected with the photogrammetric technique (PHOTOGRAMMETRIC EXAMINATION) (Figure A4);
- digital photography of the face according to the Digital Smile System (DSS) photographic protocol (EXAM DSS) (Figure A5).

### 2.4. Variables and Measurements

The obtained values from the measurements performed on the 3D virtual models created by the scanning of the plaster models have been taken as reference values.

Starting from the performed measurements:

- to assess the accuracy of each of the intraoral scanners used (Planmeca Emerald and 3Shape TRIOS) compared to the scan of the plaster model, taken as a reference virtual object;
- to assess which of the two intraoral scanners is accurate; i.e., closer to the reference values;
- to verify the accuracy of the 3D model of the face, obtained with photogrammetric technique and acquired with the cheeks apart, compared to the scan of the plaster model;
- to verify the accuracy of the 2D face photograph, obtained according to the DSS protocol with the cheeks apart, compared to the scan of the plaster model;
- to assess which of the photogrammetry and the DSS protocol is the most precise method—that is, closer to the reference values.

### 2.5. Statistical Evaluation

The statistical analysis was performed using statistical software (Prism 8.0; GraphPad Software, Inc., La Jolla, CA, USA).

The measurements made on the 3D plaster models virtual images have been taken as reference and comparison parameter. The mean and the standard deviation (DS) of the height and width sizes of the analyzed dental elements were then calculated. A T-test was used for comparing the averages calculated by pairs (for example, comparing the reference height parameter for 1.1 with the height value calculated on the TRIOS scans again for 1.1, or comparing the reference width of 1.2 with the width measured for the same element on the photogrammetric acquisitions of the face) with a significance level of 0.05 (*p* < 0.05): this method is useful in order to assess whether there was a statistically significant difference between the data obtained, comparing the different methods (Emerald, TRIOS, Photogrammetry and DSS) with the reference values.

**Figure 1:**
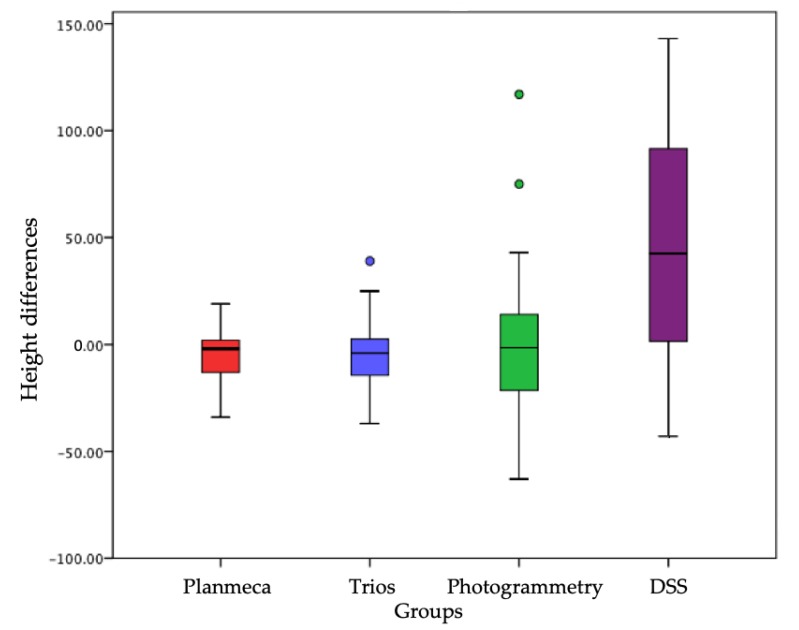
A T-test was then used for comparing the averages calculated by pairs. Differences in the distribution of height values between the different acquisition methods, each of which corresponds to a distinct sample group, with respect to the reference volumes corresponding to zero on the ordinate axis. Groups on *x* axis and height differences are on the *y* axis.

**Figure 2:**
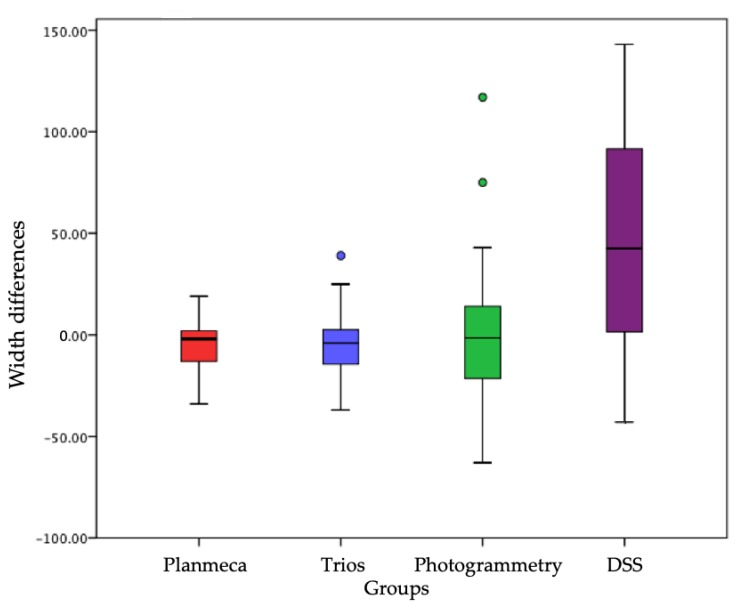
A T-test was then used for comparing the averages calculated by pairs. Differences in the distribution of mesio-distal width values between the different acquisition methods, each corresponding to a distinct sample group, with respect to the reference volumes corresponding to zero on the ordinate axis. Groups on x axis and width differences are on the y axis.

Once the average of the measurements for the sample groups was calculated, it was asked whether the difference between the means of the group was statistically significant, i.e., whether it could be said that the observed difference was not due to chance, but referred to a real difference between the group averages. For this purpose, the most effective statistical analysis is the T-test (Figure 1 and Figure 2).

The 5% significance level is frequently adopted, as it is considered that the 1/20 ratio (i.e., 0.05) is small enough to conclude that it is “unlikely” that the observed difference is due to the simple case. In fact, the difference could be due to chance, but it will be once in 20—an event that is therefore highly unlikely.

Therefore, if the zero hypothesis is rejected at the 5% significance level, then there is a 5% probability of rejecting a zero hypothesis; if the zero hypothesis is rejected at the 1% level of significance, then a 1% probability of rejecting a zero hypothesis.

## 3. Results

### 3.1. Experimental Study Results

Table 1, Table 2 and Table 3 show the values of the measurements performed with the exocad software for each patient. Values are expressed in millimeters. Each patient has an alphanumeric code shown in the first line at the top and composed of the letters PZ (*Paziente* abbreviation from Italian Patient) and a progressive numbering from 1 to 12.

The letters H and L are located below the alphanumeric code of each patient and mean height and width, respectively. Height means the linear distance measured in apico-coronal direction from the most apical point of the gingival parabola, i.e., the gingival zenith up to the incisal edge. Width is defined as the linear distance measured in the mesio-distal direction at the level of the equator of the dental elements, i.e., the maximum mesio-distal diameter level.

In the lines headed as “1.1 scanner”, “1.2 scanner” and “1.3 scanner” the values of the measurements performed on the 3D virtual models of the plaster models are shown. This dental numbering system is the one established by the World Health Organization (WHO). While for the scanner, it refers to the laboratory scanner used to create the virtual 3D model of the plaster models.

In the header lines with the labels “Emerald” and “TRIOS” are contained the measurements calculated on the 3D models of the maxillary arches, acquired through the homonymous intraoral scanners.

In the header lines with the labels “Photogrammetry” and “DSS” are reported the measurements taken, respectively, on the 3D models of the face and on the digital photographs of the patient’s face.

Due to the two-dimensional nature of digital photography it was not possible to measure the mesio-distal distance for the element 1.3, which is strongly distorted. From the comparison between the two intraoral scanners, (by evaluating the data reported in the Table 4), it emerges that their accuracy is rather overlapping, with a slight superiority in the proximity to the reference values of Planmeca Emerald compared to 3Shape TRIOS. Different measures have been obtained between different scanners and photogrammetry. In Patient 01 (PZ01 on Table 1) 1.1 tooth Exocad Height and Weight measurements have been performed; the same tooth showed a height of 6.95 mm on Planmeca Emerald Scanner, 6.67 mm on 3Shape TRIOS Scanner, and 6.47 mm on a 2D Photgrammetry. Digital Smile System protocols instead provided a 6.42 mm height. This difference could have repercussions on a definitive rehabilitation where the tolerance margins should be less than one millimeter. Despite of this data, as showed in the statistical analysis subsection, differences are not significant.

### 3.2. Statistical Evaluation

Regarding the height, the difference between the groups is not statistically significant with a *p* value > 0.05; in particular, both the Trios and the Planmeca and the photogrammetry are more precise than the DSS. Only in one case was a *p* value < 0.05, or in the comparison between the average of the reference widths for the element 1.2 and the average of the widths measured for the same element on digital photography with the DSS protocol.

It is clear that the difference between the reference values and those obtained from a 2D photograph of the face is not due to chance (there is only a 2.5% probability that it is), but it is instead due to distortion of the mesio-distal dimensions of the dental elements caused by the two-dimensional nature of a digital photograph. This mesio-distal distortion increases progressively moving from the central incisors to the posterior sectors. Table 1 shows that the difference between the groups (corresponding to the different methods of data acquisition) relative to H is not statistically significant with a *p* value > 0.05; in particular, the Trios and Planmeca are precise than both photogrammetry and DSS (Table 4).

No substantial differences emerged comparing the reference values with those obtained from the measurements conducted on intraoral scans, demonstrating how the accuracy achieved today by intraoral scanners is high.

The comparison of the photogrammetric technique and the DSS system underlines superior results in the precision of the first one, although it is reported as not statistically significant measure.

## 4. Discussion

Anamnesis and physical examination conventionally represent the preliminary phases of a dental treatment. Those steps are supported by physical impressions for the registration of the dental arches. The evaluation of the plaster models obtained from the impressions and the analysis of two-dimensional x-ray images provided complete first information of the patient’s status.

Currently, the awareness and aesthetic expectations of patients are increasing. Ror this reason, the digital aesthetic previsualization becomes a tangible expression, although virtually, of everything that the clinician could achieve, thereby legitimizing the patient’s requests and expectations [11,12,13,14,15,16,17,18].

However, it should be ensured that the pre-visualization of the treatment through the use of the virtual patient is reliable, i.e., that it allows the design of restorations dimensionally appropriate to the anatomy of the real patient. In order to verify this concept, it is necessary to evaluate the reliability of the virtual patient, defined as a set of supposable digital data on the basis of which digital treatment planning is carried out by developing the so-called virtual project.

The accuracy of the virtual patient’s data depends on the virtual rehabilitation project effectiveness (prosthetic, orthodontic, surgical). Ultimately, assessing the reliability of the virtual patient means testing the accuracy of the technological devices that allow it to be created and then represented on the computers screen.

Therefore, in this study, the accuracy of those systems applied to dental field that are currently available today for the virtual patient processing (excluding the 3D radiological techniques), were evaluated: intraoral scanners, digital photographs acquired according to a predictable protocol and repeatable as the DSS system and the most innovative tool proposed and studied here, a photogrammetric detector of the patient’s face in 3D, sometimes improperly referred as a scanner, given that it does not emit any type of laser or structured light [18,19,20,21,22,23,24].

The parameter used to compare the acquisitions obtained with these instruments was identified in the plaster models of the maxillary dental arches of the recruited patients. It has been produced by starting from physical silicone impressions that today are still considered the gold standard for an accurate recording of the dental arch morphology and for each dental element shape and size.

The study showed how the three-dimensional techniques for detecting the patient’s dental and facial features are accurate, namely the digital photography of the DSS protocol, although the statistical analysis did not report statistically significant differences.

The obviousness of this result could be disputed, especially with reference to the superiority of the photogrammetric technique compared to the 2D photography of the DSS for the analysis, not only of the dental dimensions, but also of the patient’s facial features.

The DSS photographic protocol requires less sophisticated equipment, is economically inexpensive and is normally is presented in the common dental practices. Quite different, on the other hand, are the characteristics of the photogrammetric device, the FaceShape Maxi 6 in the Maxi Line version [24,25,26,27,28].

Within the future hope of providing clinicians with an innovative approach in the diagnostic phase through a daily practice tool, such as a reflex and a tripod, it seemed useful to compare the accuracy of the two protocols—also by virtue of the repeatability and predictability of the DSS protocol, already found in the recent literature [26,27,28].

Mangano et al. [29] in their studies, demonstrated how different scanners show significant differences on trueness and precision between them. In another study, the combination of intraoral and face scans allowed to successfully restore fully edentulous patients with maxillary implant supported overdentures. Furthermore, their group of study showed how due to excellent optical properties, high mechanical resistance, restorative versatility, and different manufacturing techniques, lithium disilicate could be considered to date one of the most promising dental materials in digital dentistry. The current scanners are sufficiently accurate for capturing impressions for fabricating a whole series of prosthetic restorations (inlays/onlays, copings and frameworks, single crowns, and fixed partial dentures) on both natural teeth and implants; in addition, they could be used for smile design, and to fabricate posts and cores, removable partial prostheses and obturators. [30,31,32,33,34,35,36,37].

As for the observations related to the 2D/3D comparison, perhaps it would be necessary to emphasize that 2D and 3D measurements could coincide only if the 2D photos are taken with the plane of the photographic sensor perfectly perpendicular to the observed subject, and if the surface of the observed subject is perfectly flat. The differences instead increase if the observed surface is inclined with angles differing more and more from the 90° and how much more the shape of the same surface differs from the plane and results of the cylindrical, conical or freeform type. For this reason, if the differences on angular measurements were also examined, these could also be higher.

What instead should be emphasized in favor of 2D analyses is the quick speed and simplicity compared to all the 3D procedures. These study results can define, within the limitation related to the high differences between the 2D and 3D techniques, the field of application and the limits related to 2D techniques, offering a spot for the use of the technique in dentistry.

A final observation on the application of the photogrammetric scanner Faceshape is the possibility of obtaining the 3D scan of the entire face in 1/100th of a second, but it is not designed to scan teeth. A photogrammetric scanner for dental arches only would have a much smaller shooting field size and greater accuracy. Despite these considerations, the photogrammetric facial scanner has however provided excellent results for the measurement of individual teeth, showing great versatility of use.

Certainly, there is a superiority of photogrammetry compared to digital photography, however from the comparison between the reference values and the ones calculated on the 2D photographs, a statistically significant coherence is evident for all linear distances evaluated, except for the mesio-width distal of the upper lateral incisor for the reasons documented in the results section. Such a reduction in the accuracy of the two-dimensional DSS systematic in faithfully reproducing the mesio-distal dimensions of the dental elements of the latero-posterior sectors, which could be overcome however through a matching between digital photographs and intraoral scans, a supported operation foreseen by the DSS software, is compensated by its wide application practicality compared to the photogrammetric technique [38,39,40,41,42,43].

## 5. Conclusions

The results as already seen during the manuscript and the previous paragraphs do not show significant differences, however the reduced number of patients could influence these data. Within the limitation of the present study related to the short number of the involved patients and mainly connected to the difficulty on comparing 2D with 3D investigations, this study could be considered a starting point to carry out other researches and to definitively evaluate if there are differences between scanners and which are better, depending on the therapeutic planning.

## Figures and Tables

**Table 1:** Exocad measurements (H = height; W = Width).

Intraoral Scanners	PZ01		PZ02		PZ03		PZ04	
Dental Size	H	W	H	W	H	W	H	W
1.1 Scanner	6.98	6.09	9.48	8.23	8.81	8.11	8.30	8.08
1.2 Scanner	5.94	3.61	7.38	6.49	7.62	6.05	6.85	6.15
1.3 Scanner	9.20	7.60	9.56	7.35	9.07	7.48	7.85	7.88
1.1 Emerald	6.95	6.02	9.43	8.21	9.01	8.15	8.25	8.10
1.2 Emerald	5.92	3.75	6.52	6.50	7.23	6.14	6.74	6.53
1.3 Emerald	9.21	7.53	9.10	7.16	8.95	7.61	7.69	7.96
1.1 Trios	6.67	5.70	9.40	8.13	8.81	8.18	8.44	8.19
1.2 Trios	5.90	3.47	7.09	6.42	7.11	6.02	6.73	6.34
1.3 Trios	9.20	7.62	9.10	7.10	8.88	7.85	7.80	7.97
1.1 Photogrammetry	6.47	5.34	9.66	8.24	8.97	8.01	8.39	8.28
1.2 Photogrammetry	6.01	3.48	7.14	6.46	7.47	6.27	6.75	6.78
1.3 Photogrammetry	9.23	7.44	9.18	7.55	9.10	7.83	7.69	7.84
1.1 DSS	6.42	5.40	9.45	8.11	8.56	7.54	8.30	8.45
1.2 DSS	5.96	3.33	7.17	5.48	6.90	5.23	6.59	5.72
1.3 DSS	8.69		9.32		8.60		7.39	

**Table 2:** Exocad measurements (H = height; W = Width).

Intraoral Scanners	PZ05		PZ06		PZ07		PZ08	
Dental Size	H	W	H	W	H	W	H	W
1.1 Scanner	8.61	8.57	10.75	10.04	13.71	7.24	11.45	9.11
1.2 Scanner	7.53	7.04	7.47	7.03	13.34	6.89	9.82	7.18
1.3 Scanner	8.77	7.73	8.57	7.58	12.60	8.83	10.77	8.22
1.1 Emerald	8.66	8.77	10.75	10.24	13.43	9.14	11.40	9.12
1.2 Emerald	7.48	7.23	7.74	7.02	14.87	6.68	9.85	7.45
1.3 Emerald	8.92	7.78	8.56	7.60	13.02	9.12	10.74	8.23
1.1 Trios	8.54	8.88	10.71	10.06	13.53	9.11	11.36	9.12
1.2 Trios	7.32	7.24	7.57	7.02	14.96	6.59	9.69	7.41
1.3 Trios	8.88	7.85	8.53	7.59	13.02	8.99	10.93	8.13
1.1 Photogrammetry	8.65	8.75	10.51	8.87	13.55	9.29	11.33	9.12
1.2 Photogrammetry	7.17	7.17	7.78	7.14	13.93	6.75	9.25	7.65
1.3 Photogrammetry	8.85	7.90	8.94	7.41	12.22	8.71	10.65	8.06
1.1 DSS	8.77	8.61	10.77	9.83	13.52	9.07	11.08	9.22
1.2 DSS	7.49	5.68	7.69	5.95	14.19	5.87	9.48	6.37
1.3 DSS	8.81		8.72		12.81		10.80	

**Table 3:** Exocad measurements (H = height; W = Width).

Intraoral Scanners	PZ09		PZ10		PZ11		PZ12	
Dental Size	H	W	H	W	H	W	H	W
1.1 Scanner	10.75	8.87	9.15	8.72	10.61	8.15	9.02	8.05
1.2 Scanner	9.35	7.08	6.69	6.45	9.10	7.18	8.15	6.27
1.3 Scanner	10.34	8.70	10.35	7.69	10.02	7.87	9.18	8.09
1.1 Emerald	10.93	9.22	9.09	8.92	10.57	8.13	9.14	8.09
1.2 Emerald	9.20	7.05	6.72	6.70	9.09	7.52	8.12	6.40
1.3 Emerald	10.17	8.64	10.43	7.54	9.92	7.56	8.97	7.97
1.1 Trios	10.66	9.20	9.06	8.85	10.65	8.21	9.05	7.95
1.2 Trios	9.20	7.14	6.57	6.68	9.15	9.50	8.22	6.35
1.3 Trios	10.10	8.78	10.41	7.67	9.95	7.64	9.20	8.03
1.1 photogrammetry	10.37	9.24	9.19	8.93	10.02	7.93	9.25	8.23
1.2 photogrammetry	9.25	7.21	6.49	6.83	8.79	7.04	8.11	6.73
1.3 photogrammetry	10.04	8.87	10.58	8.22	9.91	7.44	9.43	7.86
1.1 DSS	10.74	8.89	8.61	9.15	10.26	8.23	8.33	7.98
1.2 DSS	8.72	6.04	6.17	6.03	8.68	5.75	7.64	5.58
1.3 DSS	9.74		10.20		9.69		8.82	

**Table 4:** Statistical analysis results. Results of the paired T-test with comparison of the values in pairs for each dental element (for example between dental heights of the scanned plaster models and dental heights of the intraoral scan with TRIOS relative to element 1.1). For each comparison between reference values and all other values, the *p* value is shown (H = height; W = Width).

Intraoral Scanners	1.1		1.2		1.3	
	Mean ± SD and *p*		Mean ± SD and *p*		Mean ± SD and *p*	
	H	W	H	W	H	W
Scanner	9.80 ± 1.76	8.28 ± 0.98	8.27 ± 1.96	6.45 ± 0.99	9.69 ± 1.24	7.92 ± 0.47
Emerald	9.80 ± 1.71	8.51 ± 1.02	8.29 ± 2.39	6.58 ± 0.99	9.64 ± 1.37	7.89 ± 0.55
	*p* = 0.9991	*p* = 0.5800	*p* = 0.9823	*p* = 0.7501	*p* = 0.9261	*p* = 0.8987
Trios	9.74 ± 1.76	8.47 ± 1.07	8.29 ± 2.40	6.68 ± 1.35	9.67 ± 1.36	7.94 ± 0.52
	*p* = 0.9324	*p* = 0.6623	*p* = 0.9802	*p* = 0.6390	*p* = 0.9654	*p* = 0.9348
Photogrammetry	9.70 ± 1.73	8.35 ± 1.06	8.18 ± 2.09	6.62 ± 1.05	9.65 ± 1.15	7.93 ± 0.48
	*p* = 0.8852	*p* = 0.8636	*p* = 0.9128	*p* = 0.6844	*p* = 0.9341	*p* = 0.9625
DSS	9.58 ± 1.83	8.37 ± 1.13	8.06 ± 2.20	5.59 ± 0.77	9.47 ± 1.37	
	*p* = 0.7526	*p* = 0.8308	*p* = 0.8044	*p* = 0.0254	*p* = 0.6790	

## References

[1] Yilmaz B., Abou-Ayash S. (2019). A digital intraoral implant scan technique using a combined healing abutment and scan body system. J. Prosthet. Dent..

[2] Sailer I., Muhlemann S., Fehmer V., Hammerle C.H.F., Benic G.I. (2019). Randomized controlled clinical trial of digital and conventional workflows for the fabrication of zirconia-ceramic fixed partial dentures. Part I: Time efficiency of complete-arch digital scans versus conventional impressions. J. Prosthet. Dent..

[3] Runkel C., Guth J.F., Erdelt K., Keul C. (2019). Digital impressions in dentistry-accuracy of impression digitalisation by desktop scanners. Clin. Oral Investig..

[4] De Stefano R., Bruno A., Muscatello M., Cedro C., Cervino G., Fiorillo L. (2019). Fear and anxiety managing methods during dental treatments: Systematic review of recent data. Minerva Stomatol..

[5] De Stefano R. (2019). Psychological factors in dental patient care: Odontophobia. Medicina.

[6] Patel J., Winters J., Walters M. (2019). Intraoral digital impression technique for a neonate with bilateral cleft lip and palate. Cleft Palate-Craniofacial J..

[7] Pagano S., Moretti M., Marsili R., Ricci A., Barraco G., Cianetti S. (2019). Evaluation of the accuracy of four digital methods by linear and volumetric analysis of dental impressions. Materials.

[8] Molinero-Mourelle P., Lam W., Cascos-Sanchez R., Azevedo L., Gomez-Polo M. (2019). Photogrammetric and intraoral digital impression technique for the rehabilitation of multiple unfavorably positioned dental implants—A clinical report. J. Oral Implantol..

[9] Mangano F., Mangano C., Margiani B., Admakin O. (2019). Combining intraoral and face scans for the design and fabrication of computer-assisted design/computer-assisted manufacturing (cad/cam) polyether-ether-ketone (peek) implant-supported bars for maxillary overdentures. Scanning.

[10] Kihara H., Hatakeyama W., Komine F., Takafuji K., Takahashi T., Yokota J., Oriso K., Kondo H. (2019). Accuracy and practicality of intraoral scanner in dentistry: A literature review. J. Prosthodont. Res..

[11] Cicciù M., Cervino G., Milone D., Risitano G. (2019). FEM analysis of dental implant-abutment interface overdenture components and parametric evaluation of Equator^®^ and Locator^®^ prosthodontics attachments. Materials.

[12] Cervino G., Fiorillo L., Arzukanyan A.V., Spagnuolo G., Cicciù M. (2019). Dental restorative digital workflow: Digital smile design from aesthetic to function. Dent. J..

[13] Cappare P., Sannino G., Minoli M., Montemezzi P., Ferrini F. (2019). Conventional versus digital impressions for full arch screw-retained maxillary rehabilitations: A randomized clinical trial. Int J. Environ. Res. Public Health.

[14] Cervino G., Fiorillo L., Herford A.S., Laino L., Troiano G., Amoroso G., Crimi S., Matarese M., D’Amico C., Nastro Siniscalchi E. (2018). Alginate Materials and Dental Impression Technique: A Current State of the Art and Application to Dental Practice. Mar. Drugs.

[15] Zitzmann N.U., Kovaltschuk I., Lenherr P., Dedem P., Joda T. (2017). Dental students’ perceptions of digital and conventional impression techniques: A randomized controlled Trial. J. Dent. Educ..

[16] Cicciù M., Herford A.S., Cervino G., Troiano G., Lauritano F., Laino L. (2017). Tissue fluorescence imaging (VELscope) for quick non-invasive diagnosis in oral pathology. J. Craniofacial Surgery.

[17] Sakornwimon N., Leevailoj C. (2017). Clinical marginal fit of zirconia crowns and patients’ preferences for impression techniques using intraoral digital scanner versus polyvinyl siloxane material. J. Prosthet. Dent..

[18] Rancitelli D., Cicciù M., Lini F., Fumagalli D., Frigo A.C., Maiorana C. (2017). Reproducibility of a digital method to evaluate soft tissue modifications: A study of inter and intra-operative measurement concordance. Open Dent. J..

[19] Joda T., Lenherr P., Dedem P., Kovaltschuk I., Bragger U., Zitzmann N.U. (2017). Time efficiency, difficulty, and operator’s preference comparing digital and conventional implant impressions: A randomized controlled trial. Clin. Oral Implant. Res..

[20] Joda T., Bragger U. (2016). Patient-centered outcomes comparing digital and conventional implant impression procedures: A randomized crossover trial. Clin. Oral Implant. Res..

[21] Gjelvold B., Chrcanovic B.R., Korduner E.K., Collin-Bagewitz I., Kisch J. (2016). Intraoral digital impression technique compared to conventional impression technique. A randomized clinical trial. J. Prosthodont..

[22] Gherlone E., Cappare P., Vinci R., Ferrini F., Gastaldi G., Crespi R. (2016). Conventional versus digital impressions for “all-on-four” restorations. Int. J. Oral Maxillofac. Implant..

[23] Yuzbasioglu E., Kurt H., Turunc R., Bilir H. (2014). Comparison of digital and conventional impression techniques: Evaluation of patients’ perception, treatment comfort, effectiveness and clinical outcomes. BMC Oral Health.

[24] Newby E.E., Bordas A., Kleber C., Milleman J., Milleman K., Keogh R., Murphy S., Butler A., Bosma M.L. (2011). Quantification of gingival contour and volume from digital impressions as a novel method for assessing gingival health. Int. Dent. J..

[25] Lo Giudice G., Cutroneo G., Centofanti A., Artemisia A., Bramanti E., Militi A., Rizzo G., Favaloro A., Irrera A., Lo Giudice R. (2015). Dentin morphology of root canal surface: A quantitative evaluation based on a scanning electronic microscopy study. BioMed Res. Int..

[26] Cervino G., Romeo U., Lauritano F., Bramanti E., Fiorillo L., D’Amico C., Milone D., Laino L., Campolongo F., Rapisarda S. (2018). Fem and von mises analysis of OSSTEM ^®^ dental implant structural components: evaluation of different direction dynamic loads. Open Dent. J..

[27] Bramanti E., Matacena G., Cecchetti F., Arcuri C., Cicciù M. (2013). Oral health-related quality of life in partially edentulous patients before and after implant therapy: A 2-year longitudinal study. Oral Implantol..

[28] Fiorillo L., Cervino G., Herford A.S., Lauritano F., D’Amico C., Lo Giudice R., Laino L., Troiano G., Crimi S., Cicciù M. (2018). Interferon Crevicular Fluid Profile and Correlation with Periodontal Disease and Wound Healing: A Systemic Review of Recent Data. Int. J. Mol. Sci..

[29] Mangano C., Perrotti V., Shibli J.A., Mangano F., Ricci L., Piattelli A., Iezzi G. (2013). Maxillary sinus grafting with biphasic calcium phosphate ceramics: Clinical and histologic evaluation in man. Int. J. Oral Maxillofac. Implant..

[30] Cattoni F., Teté G., Calloni A.M., Manazza F., Gastaldi G., Capparè P. (2019). Milled versus moulded mock-ups based on the superimposition of 3D meshes from digital oral impressions: A comparative in vitro study in the aesthetic area. BMC Oral Health.

[31] Mendes T.A., Marques D., Lopes L.P., Carames J. (2019). Total digital workflow in the fabrication of a partial removable dental prostheses: A case report. SAGE Open Med. Case Rep..

[32] Spielau T., Hauschild U., Katsoulis J. (2019). Computer-assisted, template-guided immediate implant placement and loading in the mandible: A case report. BMC Oral Health.

[33] Mangano F.G., Hauschild U., Veronesi G., Imburgia M., Mangano C., Admakin O. (2019). Trueness and precision of 5 intraoral scanners in the impressions of single and multiple implants: A comparative in vitro study. BMC Oral Health.

[34] Mangano C., Mangano F., Shibli J.A., Luongo G., De Franco M., Briguglio F., Figliuzzi M., Eccellente T., Rapani C., Piombino M. (2012). Prospective clinical evaluation of 201 direct laser metal forming implants: Results from a 1-year multicenter study. Lasers Med. Sci..

[35] Zarone F., Ferrari M., Mangano F.G., Leone R., Sorrentino R. (2016). “Digitally Oriented Materials”: Focus on Lithium Disilicate Ceramics. Int. J. Dent..

[36] Giuliani A., Manescu A., Larsson E., Tromba G., Luongo G., Piattelli A., Mangano F., Iezzi G., Mangano C. (2014). In vivo regenerative properties of coralline-derived (biocoral) scaffold grafts in human maxillary defects: Demonstrative and comparative study with beta-tricalcium phosphate and biphasic calcium phosphate by synchrotron radiation X-Ray microtomography. Clin. Implant Dent. Relat. Res..

[37] Cervino G., Fiorillo L., Iannello G., Santonocito D., Risitano G., Cicciù M. (2019). Sandblasted and acid etched titanium dental implant surfaces systematic review and confocal microscopy evaluation. Materials.

[38] Cervino G., Fiorillo L., Monte I.P., De Stefano R., Laino L., Crimi S., Bianchi A., Herford A.S., Biondi A., Cicciù M. (2019). Advances in antiplatelet therapy for dentofacial surgery patients: focus on past and present strategies. Materials.

[39] Cervino G., Fiorillo L., Arzukanyan A., Spagnuolo G., Campagna P., Cicciù M. (2019). Application of bioengineering devices for the stress evaluation in dentistry: the last 10 years fem parametric analysis of outcomes and current trends. Minerva Stomatol..

[40] Germano F., Bramanti E., Arcuri C., Cecchetti F., Cicciù M. (2013). Atomic force microscopy of bacteria from periodontal subgingival biofilm: Preliminary study results. Eur. J. Dent..

[41] Maiorana C., Beretta M., Grossi G.B., Santoro F., Herford A.S., Nagursky H., Cicciù M. (2011). Histomorphometric evaluation of anorganic bovine bone coverage to reduce autogenous grafts resorption: Preliminary results. Open Dent. J..

[42] Cicciù M., Cervino G., Terranova A., Risitano G., Raffaele M., Cucinotta F., Santonocito D., Fiorillo L. (2020). Prosthetic and mechanical parameters of the facial bone under the load of different dental implant shapes: A parametric study. Prostheses.

[43] Cicciù M. (2020). Prosthesis: new technological opportunities and innovative biomedical devices. Prostheses.

